# Localized retroperitoneal mass suspected malignancy: A rare case of unicentric Castleman's disease

**DOI:** 10.1002/ccr3.9451

**Published:** 2024-09-18

**Authors:** Engy S. Alhariry, Abdulkarim Hasan, Ashraf Abdelghany, Khalid Nafie

**Affiliations:** ^1^ Pathology Department Shefaa Al Orman Hospital Luxor Egypt; ^2^ Faculty of Medicine Al‐Azhar University Cairo Egypt; ^3^ Faculty of Medicine University of Granada Granada Spain; ^4^ Pathology and Laboratory Medicine Ministry of Health Khartoum Sudan

**Keywords:** Castleman's disease, histopathology, immunohistochemistry, retroperitoneal mass

## Abstract

**Key Clinical Message:**

Castleman's Disease should be considered in the differential diagnosis of retroperitoneal masses, especially in equivocal cases. Clinician should not presume all cases of retroperitoneal masses as a malignancy.

**Abstract:**

Castleman's Disease is a heterogeneous group of lymphoproliferative disorders, that can develop in lymph nodes or in extranodal sites. It has three distinct histological subtypes; hyaline vascular, plasma cell or mixed. It can be unicentric or multicentric, and sometimes oligocentric or regional. In this article, we report a case of a 30‐year‐old male who presented with a palpable left lumbar mass, clinically suspected as sarcoma vs GIST, which was surgically excised and pathologically examined revealing a rare condition of intra‐abdominal unicentric Castleman's Disease with good prognosis. Castleman's Disease should be considered in the differential diagnosis of retroperitoneal masses, especially in equivocal cases.

## 1 INTRODUCTION

Castleman's Disease is a non neoplastic, lymphoproliferative disorder that may involve lymph nodes or extranodal sites. It is classified histologically into three subtypes; hyaline vascular, plasma cell and mixed type, and according to its localization into two subtypes; unicentric and multicentric.^1^, ^2^, ^3^ The etiology of Castleman's Disease is not clear, but HHV‐8 has been attributed in many cases, particularly the multicentric plasma cell subtype.^4^ The mediastinum is the most common extranodal site, with less incidence in the head and neck region.^4^, ^5^ Mesenteric, retroperitoneal and pelvic locations are rare and infrequently reported.^4^ Unicentric Castleman's Disease has a good prognosis and mostly needs only surgery without further treatment. The patient usually persists symptomless afterward, so accurate diagnosis is essential to avoid aggressive or unnecessary treatment. The diagnosis of retroperitoneal Castleman's Disease is usually challengeable for clinicians, radiologists and even for the histopathologists, where several mimics can be considerable.^6^ Here, we present a quite infrequent case of left lumbar mass, that was suspected clinically as soft tissue sarcoma that was proven to be a Castleman's Disease on microscopic examination, aided by the immunohistochemistry.

## 2 CASE HISTORY AND EXAMINATION

A 30‐year‐old male, presented with a palpable left lumbar mass, of 2‐month duration, increasing in size. Clinically, the patient showed no clinical symptoms; however soft tissue sarcoma was suspected. His total leucocytic count was (TLC) 3 × 10^3^/mm^3^; RBCs count 4.8 mL/mm^3^, hemoglobin 13.6 g/dL, and serum LDH 357 U/L. The liver and renal function tests were within the average normal range.

## 3 METHODS

**FIGURE 1:**
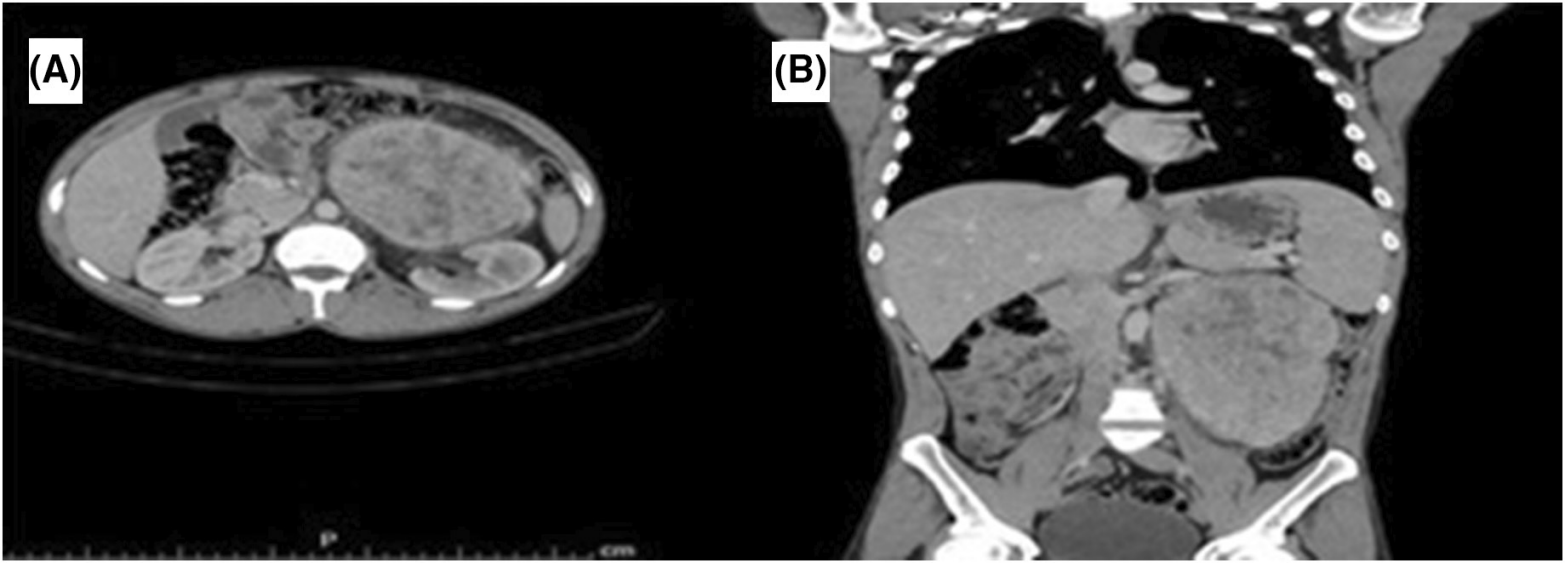
A CT scan picture showing the retroperitoneal mass.

CT examination was done and the mass was well defined, involving the left renal hilum and displacing the left kidney, with no renal, vascular or bony infiltration, and no definite bowel infiltration (Figure 1).

Surgically, the mass was found to be involving the peritoneal fat. Complete surgical excision was performed.

**FIGURE 2:**
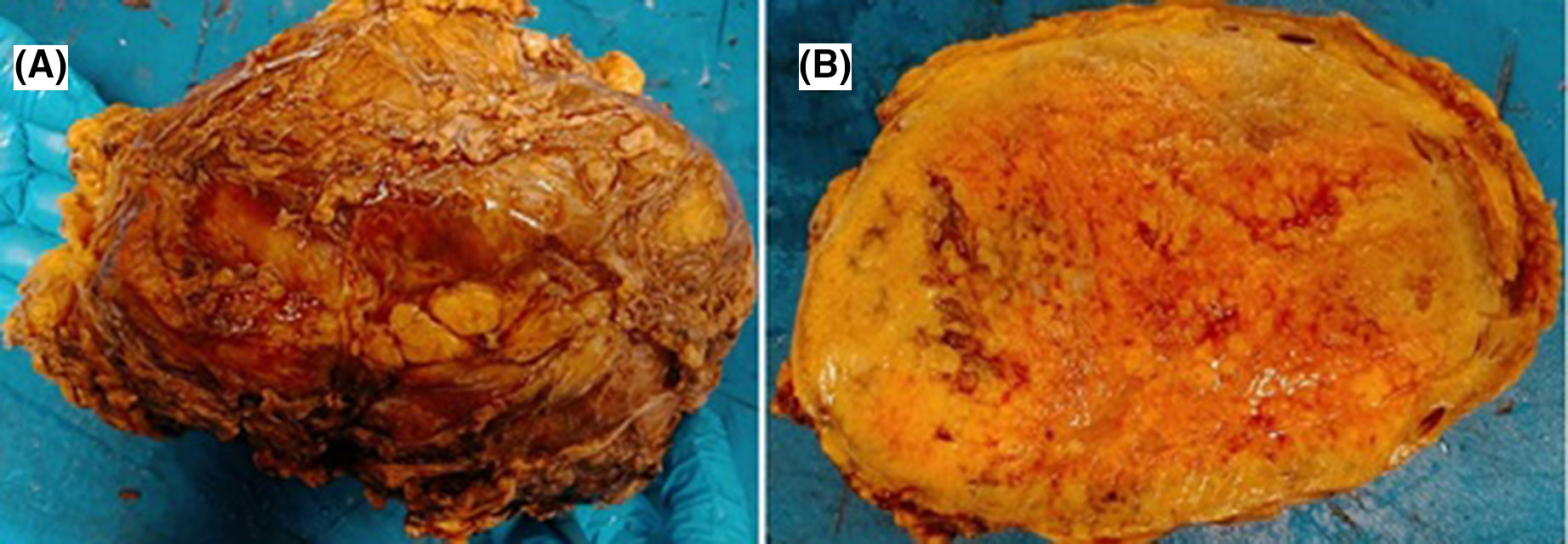
Postoperative gross picture of the excised mass.

The mass was grossly huge, well circumscribed, and measured 18 × 13 × 8 cm and weighed 350 g. The cut section was fleshy and tan, and finely lobulated (Figure 2).

**FIGURE 3:**
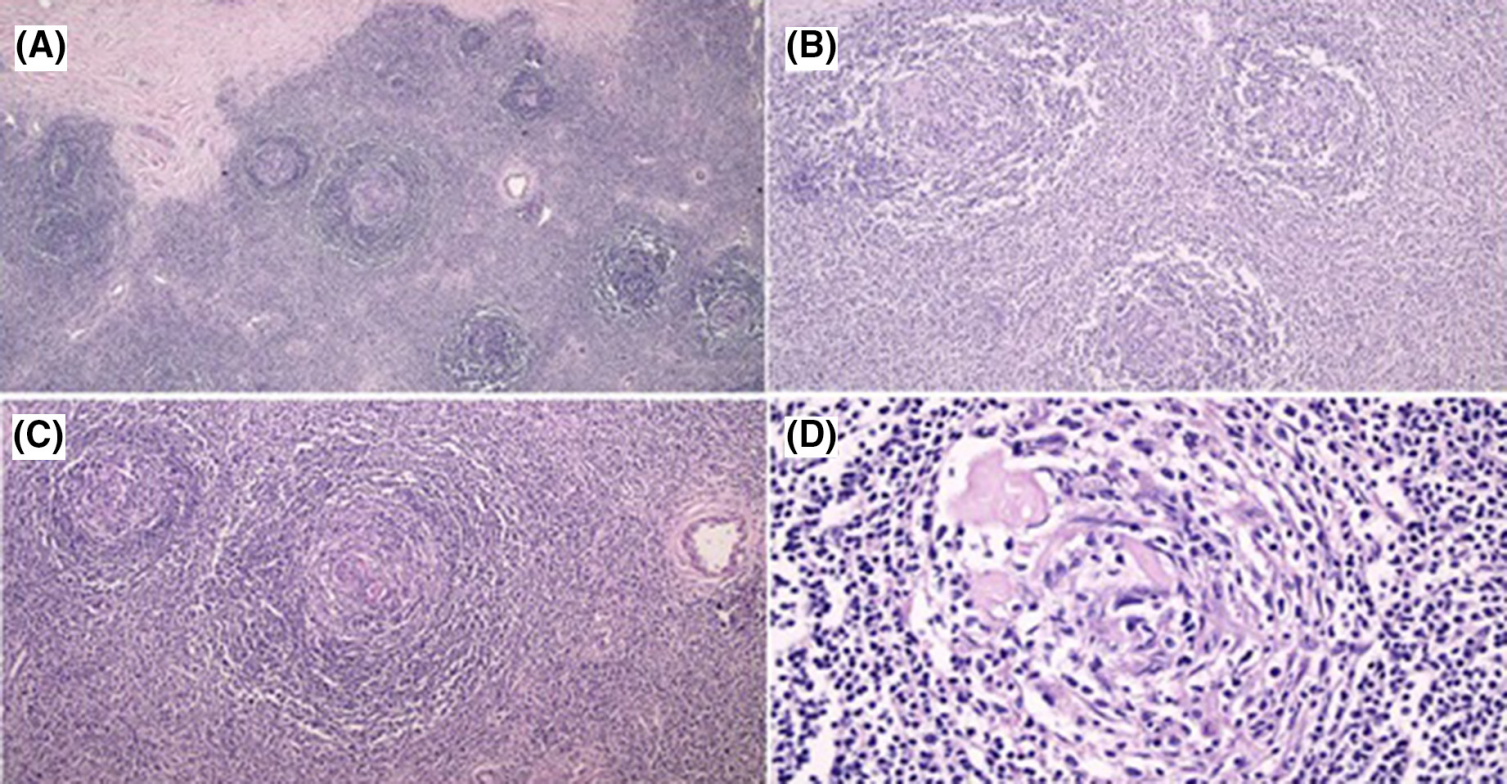
Histopathological feature of the mass showing hyperplastic follicles with intrafollicular hyalinosed vessels. Note the vessels traversing through the germinal centers (A) 40x, original magnification. (B) 100×. (C) 200×. (D) 400×.

Microscopically, it showed proliferating lymphoid follicles with vascularized germinal centers. The vessels were hyalinosed, and were penetrating into the germinal centers, with prominent surrounding hyaline material deposition, and onion skinning of the mantle zones. The stroma in between the follicles also showed many proliferating, hyalinosed vessels, with a polymorphous population of small lymphocytes and immunoblasts (Figure 3).

**FIGURE 4:**
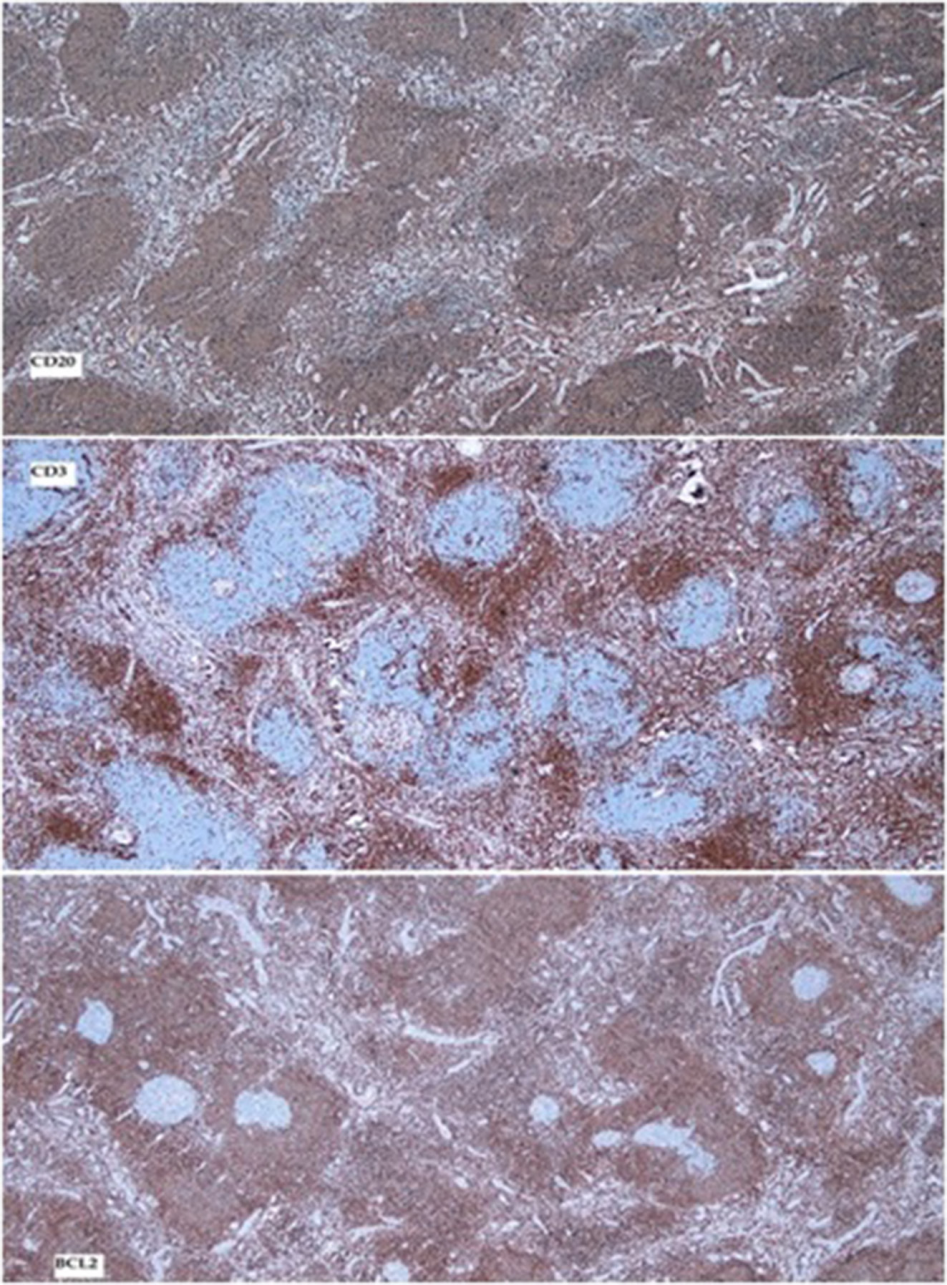
Immunohistochemistry; CD20 highlights the follicles. Note the proliferating interfollicular vessels. CD3 highlights the interfollicular T cells. BCL2 shows the expanded mantle zones and negative germinal centers.

Immunohistochemical staining was requested for determining and confirming the nature of the proliferating lymphoid cells and to exclude concomitant follicular dendritic cell sarcoma. CD20, CD3, and BCL2 were done and the results are shown in Figure 4.

## 4 CONCLUSION AND RESULTS

Unicentric Castleman's disease is the final diagnosis of this case. Though a rare clinical and histological entity, it should be included in differential diagnoses of retroperitoneal masses. It's a challenge for the clinician and pathologist to differentiate it from malignancy.

## 5 DISCUSSION

Castleman's disease is an autoimmune disorder involving hyperactivation of the human body immune system and renders multiple organ system dysfunctions. It was first described by Benjamin Castleman in the mid 1950s.^7^ The etiology remains unclear, but the disease is benign in nature and presented in localized or in generalized forms.^8^ Infection by human herpesvirus‐8 (HHV8) and HIV are associated with Castleman's disease, especially in multicenteric type and usually occurs in a younger age, and the prognosis is very poor.^9^

Unicenteric disease affects mainly mediastinum, however rarely located in the retroperitoneal region; only 7% of all cases were reported so far.^6^, ^10^ The average age of the reported cases of unicenteric Castleman's diseases is 30–40 years old, while multicenteric patients are in the age group of fifties.^6^

Castleman's disease, especially the unicenteric type, is usually asymptomatic and painless; however, masses can rapidly enlarge and compress adjacent organs, causing mass effect symptoms. Multicenteric lesions are associated with constitutional symptoms, asthenia, fever, weight loss, systemic disturbances and generalized lymphadenopathy with hepatosplenomegaly.^9^

The exact pathogenesis of Castleman's disease is still unclear, although a chronic inflammatory response seems to be the likely cause, and germinal centres of the hyperplastic lymphoid follicles produce large amounts of interleukin‐6 (IL‐6) that probably attributes to the pathogenesis of the disease.^11^, ^12^ Likewise, IL‐6 secretion is stimulated by HHV‐8 infection in case of multicenteric lesions, and several observations strongly suggest IL‐6 as the key element contributing to the disease process; (1) Following the removal of the enlarged hyperplastic lymph node, there is a reduction in the levels of serum IL‐6, acute phase reactants, gamma globulin, and an improvement in clinical condition. (2) Treatment with anti‐IL‐6 leads to relief of symptoms and signs. 3. Mice that overexpress IL‐6 exhibit a phenotype that resembles the multicenteric Castleman's disease.^12^

The unicenteric disease is characterized by the enlargement of a solitary lymph node, or at most a cluster of neighboring nodes in a singular area. The lymph nodes that are most frequently impacted are those located in the axilla, neck, abdomen, and mediastinum.^8^ The hyaline‐vascular form, which is the predominant pathogenic variety, comprises 90% of cases. This condition is characterized by the presence of swollen lymph nodes that do not cause discomfort and do not show any symptoms unless they affect nearby structures and cause compression‐related symptoms.^13^ Unicentric Castleman's disease that only affects the retroperitoneal lymph node is extremely rare. However, when it does occur, it can cause urine retention, abdominal pain, and gastrointestinal symptoms, depending on which nearby organ is affected. An increased erythrocyte sedimentation rate, C‐reactive protein, and higher white blood cell count are typically detectable on the hemogram.^14^

Histopathology is the gold standard tool for diagnosis, and immunohistochemistry can aid in ruling out sarcoma and other malignant lesions. Histological variants include hyaline vascular type, plasma cell type and mixed variant, in addition to a plasmablastic variant of multicentric diseases. The hyaline vascular type is mostly unifocal with no symptoms and diagnosed incidentally, however plasma cell type presents in multifocal lesions associated with systemic diseases.^15^ Haline vascular type, on microscopic examination, consists of lymphoid follicles with areas of hyalinization in its wall and concentric mantle lymphocytes whirls, giving an onion skin pattern, while plasma cell type contains polyclonal plasma cells with a less marked hyalinization.^9^ Due to the fact that the majority of plasma cell variant cases may exhibit hyaline vascular variant characteristics, a number of authors classify these cases as mixed type within the plasma cell variant spectrum.^16^

Differential diagnosis of plasma cell diseases / neoplasms should be considered with diseases showing marked follicular hyperplasia and/ or interfollicular plasmacytosis, such as lymphadenopathy in case of rheumatoid arthritis, plasmocytoma, lymphoplasmacytic lymphoma/Waldenström macroglobulinemia, angioimmunoblastic lymphoma and HIV‐associated lymphadenitis, and Immunohistochemistry is advised for differentiation.^17^

There are currently no established protocols for treating unicenteric Castleman's disease. However, based on the available literature, it appears that total surgical removal is an effective cure. Additionally, there have been proposals for alternative treatment methods, such as radiotherapy, yet, these options have been evaluated for potential dangers.^14^

## AUTHOR CONTRIBUTIONS

**Engy S. Alhariry:** Conceptualization; data curation; investigation; formal analysis; project administration; resources; validation; writing – original draft; writing – review and editing. **Abdulkarim Hasan:** Conceptualization; investigation; resources; software; visualization; writing – original draft; writing – review and editing. **Ashraf Abdelghany:** Conceptualization; formal analysis; software; writing – review and editing. **Khalid Nafie:** Conceptualization; resources; supervision; validation; visualization; writing – review and editing.

## FUNDING INFORMATION

This article did not receive any fund.

## CONFLICT OF INTEREST STATEMENT

All authors declare no any COI.

## ETHICS STATEMENT

Waived.

## CONSENT

Written informed consent has been obtained from the patient.

## Data Availability

With the corresponding author up on considerable request.

## References

[1] Hu S , Li Z , Wang H , et al. Clinical features and treatment outcomes of Castleman's disease in children: a retrospective cohort in China. Eur J PediatrEur J Pediatr. 2023;182(12):5519‐5530. doi:10.1007/s00431-023-05235-2 PMC10746570 37782352

[2] Nistor C , Davidescu M , Ciuche A , et al. A rare case of unicentric plasma cell type Castleman's disease in the mediastinum. Pneumologia. 2010;59(1):32‐35. PMID: 21527068. 20432791

[3] Miratashi Yazdi SA , Nazar E . Retroperitoneal Unicentric Castleman's disease, a case report. Ann Med Surg. 2022;28(79):104109. doi:10.1016/j.amsu.2022.104109 PMC9289496 35860095

[4] Pandya B , Ghosh SK , Chude G , Rajmohan MV , Narang R . Retroperitoneal Castleman's disease mimicking soft tissue tumour. Indian J Surg. 2007;69:153‐154. 23132970 10.1007/s12262-007-0009-1 PMC3452467

[5] Abdessayed N , Bdioui A , Ammar H , et al. Retroperitoneal unicentric Castleman's disease: a case report. Int J Surg Case Rep. 2017;1(31):54‐57. doi:10.1007/s12262-007-0009-1 PMC5247283 28107758

[6] Gopi P , Potty VS , Kaurav RS , Govindan K . Unicentric Castleman's disease as a localized retroperitoneal mass: a case report and review of literature. Int J Appl Basic Med Res. 2018;8(4):259‐262. 30598916 10.4103/ijabmr.IJABMR_256_17 PMC6259290

[7] Castleman B , Iverson L , Menendez VP . Localized mediastinal lymphnode hyperplasia resembling thymoma. Cancer. 1956;9:822‐830. 13356266 10.1002/1097-0142(195607/08)9:4<822::aid-cncr2820090430>3.0.co;2-4

[8] Lu ZH , Wu M . Localized Castleman's disease of plasma cell type in the abdomen. Chin Med J. 2011;124:2789‐2791. 22040445

[9] Cesarman E , Knowles DM . The role of Kaposi's sarcoma‐associated herpesvirus (KSHV/HHV‐8) in lymphoproliferative diseases. Semin Cancer Biol. 1999;9:165‐174. 10343068 10.1006/scbi.1998.0118

[10] Vora K , Dash A , Bach A , Gopalan A , Russo P . A case of Castleman's disease that presented as a retroperitoneal mass. Nat Clin Pract Urol. 2007;4:285‐288. 17483814 10.1038/ncpuro0800

[11] Shrestha AL , Mishra A , Khadka S , Dhakhwa R . Retroperitoneal Castleman's disease in a young Nepalese girl: a rare cause of childhood abdominal mass. Ann Med Surg. 2024;86(2):1080‐1084. doi:10.1097/MS9.0000000000001579 PMC10849377 38333308

[12] Yoshizaki K , Matsuda T , Nishimoto N , et al. Pathogenic significance of interleukin‐6 (IL‐6/BSF‐2) in Castleman's disease. Blood. 1989;74:1360‐1367. 2788466

[13] Wang S , Chen S , Xu J , Cai S . Clinicopathological characteristics of unicentric retroperitoneal Castleman's disease: a study of 14 cases. World J Surg Oncol. 2015;14:3. 10.1186/s12957-015-0756-6 PMC4704256 26739518

[14] Talat N , Belgaumkar AP , Schulte K‐M . Surgery in Castleman's disease: a systematic review of 404 published cases. Ann Surg. 2012;255:677‐684. 22367441 10.1097/SLA.0b013e318249dcdc

[15] de Vries IA , van Acht MM , Demeyere T , et al. Neoadjuvant radiotherapy of primary irresectable unicentric Castleman's disease: a case report and review of the literature. Radiat Oncol. 2010;5:7. 20122250 10.1186/1748-717X-5-7 PMC2827478

[16] Sevilla‐Lizcano DB , Frias‐Soria CL , Ortiz‐Hidalgo C . Enfermedad de Castleman. Análisis histopatológico e inmunohistoquímico de treinta y nueve casos [Castleman disease. Histopathological and immunohistochemical analysis of 39 cases]. Gac Med Mex. 2017;153(5):550‐558. doi:10.24875/GMM.17003021 29099112

[17] An Rhee F , Stone K , Szmania S , et al. Castleman's disease in the 21st century:an update on diagnosis, assessment, and therapy. Clin Adv Hematol Oncol. 2010;8:486‐498. 20864917

